# Endoscopic and Surgical Management of a Hayes Type III-G Cystic Duct Anomaly Causing a Mirizzi Type I Syndrome

**DOI:** 10.1155/1998/97313

**Published:** 1998

**Authors:** Paul G. Anderson, James Toouli, Thomas G. Wilson, Michael Graham

**Affiliations:** Gastrointestinal Surgical Unit Department of Surgery Flinders Medical Centre The Flinders University of South Australia Australia


*HPB Surgery*, 1998, Vol. 10, pp. 399-402
Reprints available directly from the publisher
Photocopying permitted by license only

© 1998 OPA (Overseas Publishers Association)
Amsterdam B.V. Published under license
under the Harwood Academic Publishers imprint,
part of The Gordon and Breach Publishing Group.
Printed in India.

### *Case Report*

### Endoscopic and Surgical Management of a Hayes Type III-G Cystic Duct Anomaly Causing a Mirizzi Type I Syndrome

PAUL G. ANDERSON[a], JAMES TOOULI[a,*], THOMAS G. WILSON[a] and MICHAEL GRAHAM[a]

[a]*Gastrointestinal Surgical Unit, Department of Surgery, Flinders Medical Centre, The Flinders University of South Australia*

*(Received 8 August 1996)*

*Keywords:* Mirizzi Syndrome, Hayes bile duct, anomalies, endoscopic stent

### INTRODUCTION

A Mirizzi Type I syndrome usually occurs as the result of gallstones impacting in the cystic duct or Hartmann's pouch and then causing external compression of the common hepatic duct. It is described as an infrequent cause of obstructive jaundice occurring in only 0.7-1.1% of cholecystectomies.

In this case report, we present a case of Mirizzi Syndrome associated with an impacted calculus in the gallbladder, an absent cystic duct (Hayes Type II-G anomaly) and a coincidental common bile duct stone. ERC diagnosed the syndrome, defined the anatomical variation and through interim stenting allowed the patient to recover from her obstructive jaundice and sepsis and to undergo open cholecystectomy safely.

### CASE REPORT

A 65 year old female presented with a four-day history of abdominal pain and deepening jaundice. The day prior to admission she developed fever and rigors. Examination confirmed a deep jaundice, moderate to severe dehydration and tenderness in the right upper quadrant.

The blood pressure was 100/60 and she had a pulse of 100/min. Her temperature was 38°C, white cell count 4.7 ($\times 10^9$/L). Platelets 84 ($\times 10^9$/L), INR 1.1, APTT 27. Urea was 17.8 mmol/l and Creatinine 0.147 mmol/l. Liver function tests; AP 97 U/L, GGT 260 U/L, ALT 45 U/L, AST 35 U/L, Bilirubin 245 $\mu$mol/l and a normal amylase.

*Correspondence: Professor JAMES TOOULI Head – Gastrointestinal Surgical Unit Department of Surgery, Flinders Medical Centre, Bedford Park, Adelaide, South Australia, Australia. Tel: 61 8 8204 5213, Fax: 61 8 8204 5966, E-mail: Jim.Toouli @ flinders, edu.au.

A diagnosis of ascending cholangitis was made with potential disseminated intravascular coagulopathy (DIC). The patient was resuscitated with both crystalloids and colloids. Broad spectrum antibiotics were started and 6 packs of platelets were given.

Ultrasonography demonstrated a single stone in the gallbladder without any sonographic evidence of cholecystitis. The bile duct was measured at 10 mm. The cause of patient's jaundice was not defined by ultrasound and she underwent ERCP within 24 hours.

ERC demonstrated a 5 mm stone in the common bile duct. Cholangiography confirmed the stone in the gallbladder, a very short cystic duct and a dilated common hepatic duct. Intrahepatic ducts filled poorly. The stone in the bile duct was thought to be the cause of the clinical presentation. A 1.5 cm sphincterotomy was done and the stone removed from the bile duct.

The patient did not improve. Her bilirubin remained elevated and her liver function tests deteriorated further. A second ERC was done. The sphincterotomy was noted to be wide open. A stone was noted impacting the common hepatic duct from the gallbladder (Fig. 1).

Initial attempts to pass a guide wire beyond the calculus failed. However, using an endotorque guidewire (Wilson Cook), the proximal biliary duct was negotiated. A catheter was then passed over the wire allowing contrast into the proximal biliary duct demonstrating the cause of the obstruction. A 7 F 12 cm stent was inserted across the narrowed duct and free flow of obstructed bile was noted (Fig. 2).

The patient made a rapid recovery, her jaundice receding and her LF T's returning to normal. The patient remained in hospital 5 days following the placement of the stent. Two months later she was readmitted, for elective cholecystectomy. It was decided to remove the stent prior to the operation, however on removing it the stone in the gallbladder again obstructed the CBD, hence a new stent was reinserted. The patient then underwent an open cholecystectomy 3 days later, during the same admission.

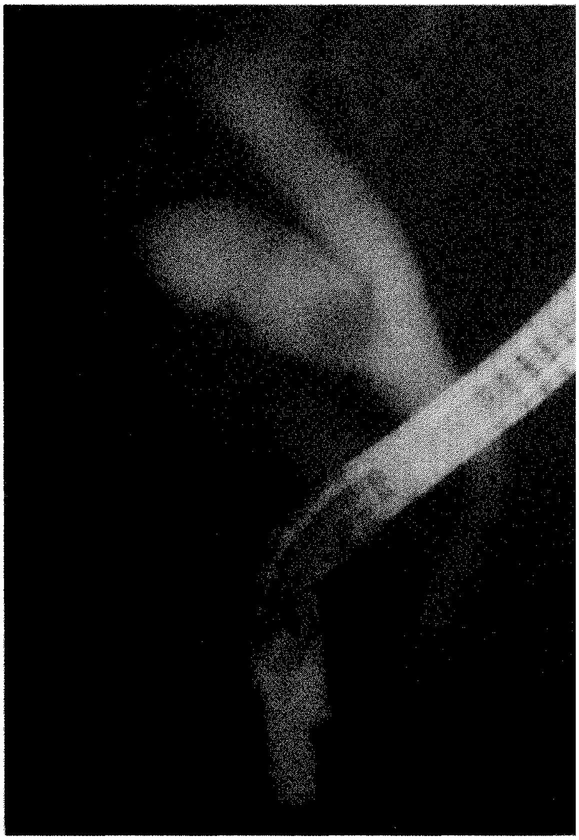

FIGURE 1 Impaction of gallstone into the common hepatic duct.

At operation a Hayes Type III-G abnormality was confirmed. There was no cystic duct, just a large calibre opening into the common bile duct. The gallbaldder was fibrosed and Calot's triangle was ill defined. Acute on chronic inflammation made a retrograde dissection necessary. The cystic duct artery was noted branching directly off the right hepatic artery. This was ligated in continuity and divided. During dissection of the gallbladder and in Calot's triangle, the stent in the bile duct was invaluable in providing a palpable guideline for the position of the bile duct. Due to the absence of a definable cystic duct, a distal rim of 0.5 cm of gallbladder was left attached to the common bile duct which was then oversewn with 3.0 Maxon. The stent was left in situ in order to facilitate bile drainage into the duodenum and prevent any bile leakage from the cystic duct stump.

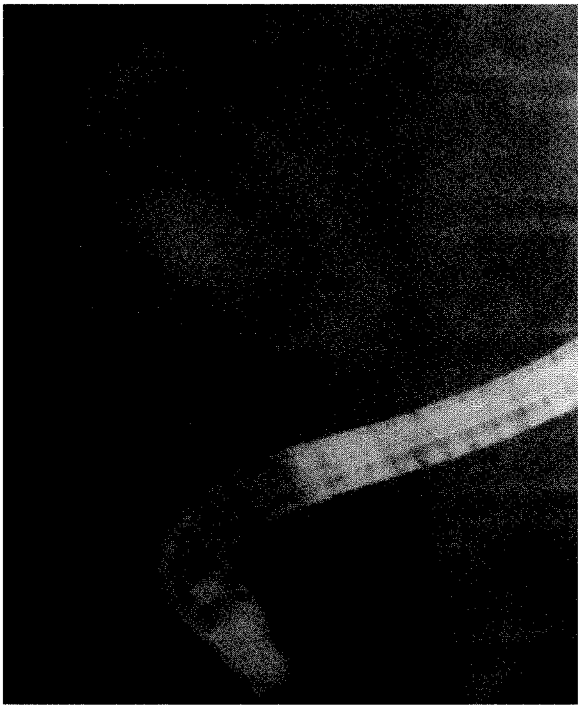

FIGURE 2 Gallstone disimpacted and stent placed allowing biliary drainage.

The patient made an uneventful recovery and her stent was removed endoscopically at 1 month. At 12 months follow up there have been no further problems.

### DISCUSSION

Obstructive jaundice caused by a stone in the cystic duct or Hartmann's pouch is known as Mirizzi's Syndrome [1]. Mirizzi first described a functional hepatic syndrome. He listed the following as aetiological causes

(1) an anatomic variation of the cystic duct or neck of the gallbladder adjacent to the common hepatic duct;
(2) impaction of a stone in the cystic duct or neck of gallbladder;
(3) partial mechanical obstruction of the common duct by the stone or by the resulting inflammatory reaction and oedema;
(4) jaundice, recurrent cholangitis and if longstanding, cholangitic cirrhosis [2].

McSherry [3], redefined the Mirizzi Syndrome in 1982. Type I occurs when there is external compression of the common hepatic duct by a large stone impacted in the cystic duct or Hartmann's pouch. In Type II, a cholecystocholedochal fistula is present caused by a calculus which has eroded partly or completely into the common hepatic duct.

Hayes [4], reviewed 400 biliary operations and reported biliary anomalies in 189. These he divided into four major types. Type I, accessory ducts; Type II, anomalous length of common hepatic duct; Type III, anomalous junction of cystic with common hepatic duct; Type IV, anomaly of common duct. The Type III anomaly, into which this patient belongs, constitutes only 6 % of total anomalies in Hayes' analysis. These are further subdivided alphabetically, Type III-G being an absent cystic duct.

The patient in this report was an example of Mirizzi Type I with a Hayes Type III G anomaly. However, it is unusual for a number of reasons. At the time of presentation, ultrasound was equivocal about dilatation of extrahepatic ducts, a stone was found in the CBD and presumed to be the cause of obstruction. However, despite its removal the patient did not improve. At the second ERC, the common hepatic duct was dilated and the effect of the stone in the gallbladder in causing a Mirizzi syndrome became evident. In addition, the anomalous biliary anatomy was defined and later confirmed at surgery.

The use of an endoscopic biliary stent in the management of a Mirizzi Syndrome has been previously described [5] and provides acute relief of the obstruction whilst the patient is resuscitated for subsequent definitive treatment. Typically, Mirizzi syndrome produces severe, acute or chronic changes within Calot's triangle. It is generally regarded that this syndrome is a contraindication to laparoscopic cholecystectomy.

Open cholecystectomy can also be difficult, as it was in this case where chronic inflammation had eliminated normal anatomical boundaries. We believe the insertion of a biliary stent was a useful adjunct to open surgery. It provided a palpable guide to the anatomical position of the bile duct, making dissection in Calot's triangle less hazardous.

Anatomical variations of the biliary tree are reported in 0.7–1.1 % of patients undergoing cholecystectomy [6, 7] and the Mirizzi Syndrome also is infrequently encountered [8]. In this case, the use of an endoscopic stent provided acute relief in an acutely ill patient and allowed for the subsequent elective approach to treatment. After the second ERC we planned to remove the stent and then proceed to cholecystectomy. However, the obstruction recurred immediately due to the anomalous anatomy in this patient. Consequently this patient needed to have her gallbladder operation with the endoscopic stent in situ. At operation, the presence of the stent proved to be of assistance in helping to define the anatomy. The stent was removed as a day procedure once the patient had recovered from the open operation.

This case highlights the combination of an endoscopic and operative approach in the treatment of gallstones associated with an anatomical anomaly which led to cholangitis. An endoscopic approach was used prior to cholecystectomy so that at the time of surgery the patient was neither jaundiced nor had acute cholangitis.

Data from previously published studies [9, 10, 11] support an initial endoscopic approach in the treatment of patients with cholangitis as the morbidity and mortality is significantly reduced when compared to a direct open operation. Once the acute illness has subsided it allows for treating such patients subsequently via either a laparoscopic or as in this patient via open cholecystectomy.

### Acknowledgements

Amanda Sowter for her secretarial assistance in the preparation of this case report.

### References

[1] Mirizzi, P. L. (1948). Sindrome del conducto hepatico. *J. Int. Chir.*, **8**, 731.

[2] Starling, J. R. and Matallana, R. H. (1990). Benign mechanical obstruction of the common hepatic duct (Mirizzi Syndrome). *Surgery*, **88**, 737–40.

[3] Dewar, G. (1990). Operative strategy in the Mirizzi Syndrome. *Surgery. Gynecol. Obstet*, **171**, 157–9.

[4] Hayes, M. A., Goldenberg, J. S. and Bishop, C. C. (1958). The developmental basis for bile duct anomalies. *Surg. Gynecol. Obstet*, **107**, 447–456.

[5] McSherry, C. K., Ferstenberg, H. and Virshup, M. (1982). The Mirizzi Syndrome: suggested classification and surgical therapy. *Surg. Gastroenterol*, **1**, 219–25.

[6] Blumgart, L. H. (1988). Surgery of the Liver and Biliary Tract. (Ed. H. Blumgart) Edinburgh, Churchill Livingstone.

[7] Bower, T. C. and Nagorney, D. M. (1988). Mirizzi Syndrome. *HPB Surgery*, **1**, 67–76.

[8] Morelli, A., Narducci, F. and Ciccone, R. (1978). Can Mirizzi Syndrome be classified into acute and chronic form? *Endoscopy*, **10**, 109.

[9] Worthley, C. S. and Toouli, J. (1990). Endoscopic decompression for acute cholangitis due to stones. *ANZ. J.* Surg., **60**, 355–359.

[10] Leese, T., Neoptolemos, J. P., Baker, A. R. and Carr-Locke, D. L. (1986). Management of acute cholangitis and the impact of endoscopic sphincterotomy. *Br. J. Surg.*, **73**, 988–992.

[11] Lai, E. C. S., Mok, F. P. T., Tan, E. S. Y. *et al.* (1992). Endoscopic biliary drainage for acute cholangitis. *N. Eng. J. Med.*, **326**, 1582–1586.

